# A Single Bout of Exercise Improves Motor Memory

**DOI:** 10.1371/journal.pone.0044594

**Published:** 2012-09-04

**Authors:** Marc Roig, Kasper Skriver, Jesper Lundbye-Jensen, Bente Kiens, Jens Bo Nielsen

**Affiliations:** 1 Department of Neuroscience and Pharmacology, University of Copenhagen, Copenhagen, Copenhagen, Denmark; 2 Department of Exercise and Sport Sciences, University of Copenhagen, Copenhagen, Denmark; University of Milan, Italy

## Abstract

Regular physical activity has a positive impact on cognition and brain function. Here we investigated if a single bout of exercise can improve motor memory and motor skill learning. We also explored if the timing of the exercise bout in relation to the timing of practice has any impact on the acquisition and retention of a motor skill. Forty-eight young subjects were randomly allocated into three groups, which practiced a visuomotor accuracy-tracking task either before or after a bout of intense cycling or after rest. Motor skill acquisition was assessed during practice and retention was measured 1 hour, 24 hours and 7 days after practice. Differences among groups in the rate of motor skill acquisition were not significant. In contrast, both exercise groups showed a significantly better retention of the motor skill 24 hours and 7 days after practice. Furthermore, compared to the subjects that exercised before practice, the subjects that exercised after practice showed a better retention of the motor skill 7 days after practice. These findings indicate that one bout of intense exercise performed immediately before or after practicing a motor task is sufficient to improve the long-term retention of a motor skill. The positive effects of acute exercise on motor memory are maximized when exercise is performed immediately after practice, during the early stages of memory consolidation. Thus, the timing of exercise in relation to practice is possibly an important factor regulating the effects of acute exercise on long-term motor memory.

## Introduction

It is well established that physical activity has a positive impact on cognition and brain function [1]. Regular aerobic exercise, in particular, produces moderate yet robust improvements in the performance of some cognitive tasks, especially in those involving attention, processing speed and executive functioning [2]. Along with these behavioural improvements, aerobic exercise has also been shown to promote both functional and structural adaptations in the human brain [3]. Examples of these adaptations include increases in brain activation [4], blood flow [5], connectivity [6] as well as brain volume in areas such as the hippocampus [7] and frontal and parietal cortices [8]. Physical activity programs including aerobic exercise may also confer neuroprotective effects, thereby reducing the risk of age-related cognitive decline [9] and the progression of neurological conditions such as Alzheimer's [10] and Parkinson's disease [11], [12] as well as accelerating recovery after stroke [13], [14]. Thus, there is mounting evidence that regular physical activity, and aerobic exercise in particular, is an effective yet inexpensive strategy to maintain a healthy cognitive status, improve recovery after brain injury and reduce the incidence of some age-related neurodegenerative diseases.

Rather than focusing on the long-term effects of periodic bouts of aerobic exercise, other studies have explored the immediate effects that a single bout of exercise may have on cognitive performance [15]–[17]. The rationale behind the use of acute exercise is usually founded upon psychological and biological grounds. Psychological theories postulate that an adequate level of exercise-induced arousal [18] may optimize the allocation of mental resources and thus facilitate cognitive processing [19] and consolidation of memory [20]. More biological perspectives [21], in contrast, attribute the benefits of acute exercise on cognition to an increased concentration of neurochemicals such as epinephrine [22] and brain derived neurotrophic factor (BDNF) [23] during exercise, which facilitate memory consolidation and learning. The results of studies analyzing the immediate effects of exercise on cognition, however, are equivocal, with some studies reporting moderate effects while others showing no, or even detrimental effects [24], [25]. Conflicting results might be explained from differences in the characteristics of exercise, when the cognitive task was performed in relation to exercise, when performance was measured, types of subjects studied and the type of cognitive function assessed [24], [26], [27].

The majority of studies assessing the immediate effects of exercise on brain function have focused on low-level processing cognitive functions measured through neuropsychological tasks such as simple and choice reaction time [15], [16], [19] involving discrete movements (e.g. finger tapping). In contrast, much less attention has been placed on other tasks involving complex movements, requiring high-level executive functions, which demand the integration of information from different brain areas. For example, only a small number of studies have investigated the acute effects of exercise on specific forms of memory [17], [28]–[32] and learning [33], and none of them specifically the effects on motor memory consolidation and motor skill learning. Furthermore, with few exceptions [29], [33], [34], the majority of cognitive studies have focused on improvements in performance during practice (i.e. acquisition) or shortly after (e.g. short-term retention) [17], [28]–[32] rather than on the long-term effects of acute exercise on memory (i.e. long-term retention), which is perhaps a more representative measure of memory consolidation and learning [35]. It is therefore uncertain if the potential benefits that acute exercise may have on some types of learning modalities can be extrapolated to other, more complex tasks involving other learning paradigms such as motor skill learning and whether these benefits can optimize the formation and long-term retention of motor memory.

Motor skill learning is of vital importance during different periods of the lifespan. In addition to childhood development, it is of particular importance during rehabilitation, when strategies to optimize the acquisition and retention of motor skills become critical to stimulate the recovery of motor function. We examined whether acute exercise can be used as a strategy to optimize motor skill learning in healthy young subjects. Specifically, we investigated whether an intense bout of cycling exercise could optimize the acquisition and retention of a motor skill. In addition, we explored if the timing of the exercise in relation to the practice of the motor task had any influence on motor memory and skill learning. This was assessed by comparing the acquisition and retention of a motor skill when the bout of exercise was performed either before (PRE) or after (POST) practicing a visuomotor accuracy-tracking task (AT) or after rest (CON). Motor skill acquisition was assessed during practice and retention was measured 1 hour, 24 hours and 7 days after practice.

We hypothesized that the performance of a single bout of exercise before motor practice (PRE) would improve the acquisition of the motor skill and that exercise after practice (POST) would mainly optimize its retention [33]. Our results, however, revealed that acute exercise did not have any significant effect on the rate of skill acquisition. In contrast, regardless of the timing of exercise, participants engaging in acute exercise showed an overall better long-term retention of the motor skill in comparison to the non-exercise group (CON). In addition, the group that exercised after practicing the AT (POST) showed a better long-term retention of the motor skill than the group that practiced the AT after exercise (PRE).

## Materials and Methods

### Ethics statement

The ethics committee for the Greater Copenhagen area approved the study and all subjects gave written informed consent prior to participation. The study was performed in accordance with the declaration of Helsinki II.

### Subjects

Forty-eight right-handed healthy young male subjects were recruited to participate in the study. All subjects were naïve to the AT before enrolment. Exclusion criteria for participation were: age below 18 or above 35, body mass index (BMI) above 30, self-reported history of neurological, psychiatric or medical diseases as well as current intake of medications and/or recreational drugs affecting the central nervous system and/or the ability to learn. Subjects were randomly assigned to one of the three groups that performed the AT either before (POST) or after (PRE) an acute bout of intense cycling exercise, or after rest (CON). The randomization was stratified to ensure that the three groups were matched for age, as well as fitness level (Table 1) as measured by their peak oxygen consumption (VO_2peak_) in a graded exercise protocol (see pre-examination) because these factors have been reported to modulate the effects that acute exercise has on cognitive performance [36], [37].

**Table 1:** Characteristics of subjects.

	CON	PRE	POST
Age (years)	23.93 (20–32)	24.06 (21–33)	24.37 (20–30)
Height (m)	1.84 (1.75–1.90)	1.83 (1.71–1.90)	1.84 (1.72–1.91)
Weight (kg)	76.62 (71.60–95.70)	76.80 (63–88.60)	76.71 (65–89)
BMI (Kg/m^2^)	22.52 (20.08–25.75)	22.61 (20.10–25.89)	22.54 (20.58–24.69)
VO_2_ peak	52.76 (45.2–66.40)	53.77 (44.60–64.10)	52.93 (44.1–62.2)

Groups were matched for age and aerobic fitness (VO_2_ peak). Data are presented as means and ranges.

### Pre-examination

At least 48 hours before the main experiment subjects reported to the laboratory to undertake a graded exercise protocol on a cycle ergometer (Ergomedic 839 E, Monark, Sweden). The exercise protocol was used to determine their VO_2peak_ as well as their blood lactate concentration at different intensity levels. Exercise started with a 5 min warm-up at a workload of 75W. After warm-up, the workload of the cycle ergometer was set at 100W and then gradually increased by 50W every 3 min until exhaustion. Subjects were instructed to maintain a pedalling rate above 70 rpm throughout the entire protocol and exhaustion was defined as the point at which subjects could not consistently maintain this pedalling rate. Pulmonary ventilation, heart rate (HR), oxygen consumption (VO_2_), exhalation of carbon dioxide (VCO_2_) and respiratory exchange ratio (RER) were registered every 15 sec by an on-line gas analyzing system (MasterScreen CPX®, Carefusion, Germany). The criteria used to ascertain that subjects reached their VO_2peak_ was a levelling off of VO_2_ with increasing workload and a RER above 1.14 [38]. Capillary blood samples from the fingertips of the non-dominant hand were drawn with disposable diabetic lancets (Accu-check Safe-T-Pro Plus®, Roche Diagnostics, Switzerland) at baseline, at the end of each 3 min block as well as 5 and 10 min after exhaustion. Blood lactate levels were analyzed with a hand-portable lactate analyzer (Accutrend Plus System®, Roche Diagnostics, Switzerland), which has shown good accuracy and reliability [39]. A Borg scale [40], ranging from 6 (no exhaustion) to 20 (complete exhaustion), was used to record the subjective level of perceived exertion at the end of each 3 min block.

### Design

**Figure 1:**
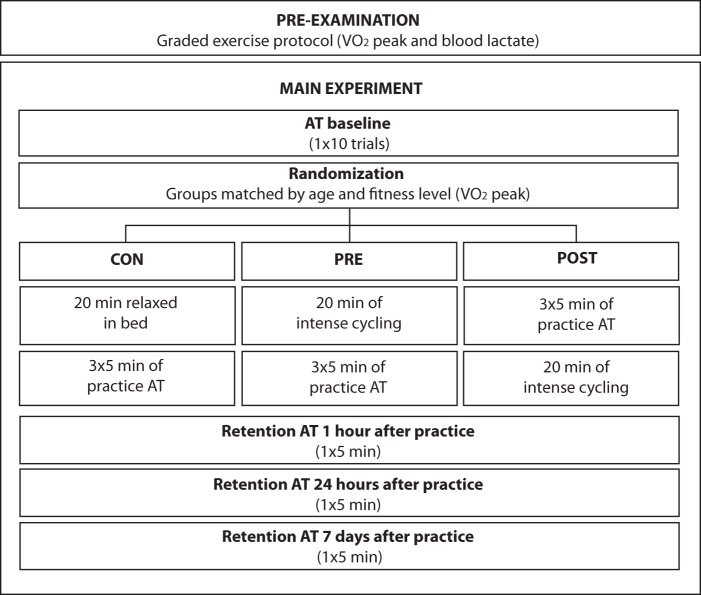
Phases of the experiment.

In addition to the pre-examination visit, subjects had to report to the laboratory on three other occasions. Figure 1 shows the different phases of the experiment. On the first day subjects performed 10 trials of the AT to measure performance at baseline. After the baseline measurement subjects in the CON group were instructed to rest in bed for 20 min whereas subjects in the PRE group undertook a 20 min intense cycling exercise protocol. After the resting period (CON) or the performance of the exercise bout (PRE) subjects practiced the AT. Subjects in the POST group practiced the AT first and then undertook 20 min of intense cycling. After practice, subjects in the CON and PRE groups were placed in a hospital bed where they rested for 1 hour. To ensure that all groups had the same resting time (1 hour) between the end of practice and the first retention test the POST group rested 40 min after exercise. To minimize changes in arousal and interferences in the consolidation of memory during the resting period, subjects were not allowed to listen to music nor sleep but were allowed to read. Improvements in performance during practice measured the rate of motor skill acquisition. To evaluate the impact of exercise on the short-term retention of the motor skill, a retention test of the AT was performed after the resting period (1 hour). The impact of exercise on the long-term retention was assessed with a retention test 24 hours and 7 days after practicing the AT.

### Exercise

The exercise groups performed an exercise protocol of the same intensity on a cycle ergometer (Ergomedic 839 E, Monark, Sweden). Cycling was chosen to minimize fatigue in the upper limb and because it appears to be more effective in improving cognitive performance than treadmill running [24]. The exercise protocol was specifically designed to ensure high levels of blood lactate concentration (≥10 mmol/l) prolonged after exercise [33] while limiting excessive fatigue and dehydration [26]. This relatively high intensity was used because intensity appears critical to optimize memory and learning [33]. Briefly, the protocol started with 2 min of warm-up at 75W followed by 3×3 min blocks of high intensity interspersed with 3×2 min blocks of low intensity (50W) in between. During the last 3 min of the exercise protocol subjects were allowed to rest. The workload during the blocks of high intensity was adjusted to each subject's workload based on the results obtained in the graded exercise test. Heart rate, measured by telemetry (WearLink 31 transmitter, Polar Electro, Kempele, Finland) was used to monitor the intensity of the exercise. To ensure that the exercise had reached sufficient intensity (≥10 mmol/l), a blood sample (2 ml) was drawn from the cubital vein of the non-dominant arm immediately after exercise and blood lactate concentration was assessed with a blood gas analyzer (ABL800 Flex analyzer, Radiometer Medical ApS, Denmark). To evaluate the potential influence of fatigue a Borg scale [40] was used to record the level of perceived exertion at the end of exercise.

### Visuomotor accuracy-tracking task (AT)

**Figure 2:**
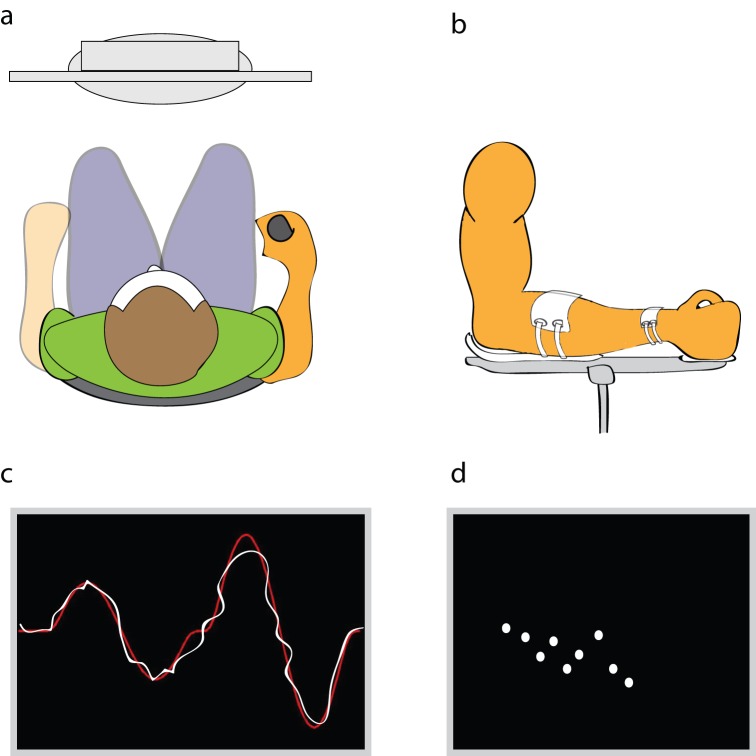
The visuomotor accuracy-tracking task (AT). (**a**) Subjects were seated in front of a computer screen with the right forearm placed in a molded rigid custom-made arm support. (**b**) The forearm was positioned in a neutral semi-prone position. (**c**) The main application window contained a target consisting of a fixed double sine wave curve (red) that subjects had to track with the torque signal (white). (**d**) The second window provided visual feedback on the performance of the AT task by displaying a series of white dots on a coordinate axis.

To perform the AT, subjects were comfortably seated on a 65 cm stool in front of a computer screen with the right arm placed in a molded rigid custom-made arm support (Fig. 2A). The shoulder joint was held in a neutral position and the elbow joint angle was positioned at 100–110° of flexion. The forearm, firmly strapped to the arm support, was positioned in a neutral semi-prone position (Fig. 2B). The distal part of the arm support consisted of a handle with the rotational axis located coaxially with the axis of rotation of the wrist. The handle was equipped with a built-in potentiometer connected to a strain gauge transducer, which provided information on the torque applied to the handle when muscle isometric contractions involving wrist extension or flexion were applied. The wrist torque signal during tracking was amplified with a 600 Hz carrier frequency amplifier for strain gauge transducers (ME30, HBM, Germany), sampled at 100 Hz and stored on a computer via a data acquisition interface (Micro1401, CED, UK) for off-line analysis.

A software application built on Labview® (National Instruments, USA) was created to design the AT. The application displayed two windows on a computer screen. The main window contained a target consisting of a fixed double sine wave curve (Fig. 2C) that the subjects had to track, as accurately as possible, by applying wrist extension and flexion isometric contractions. The amplitude of the target trajectory (y-axis) was adjusted to approximately <5% of the maximal voluntary contraction of wrist extensors. Briefly, the isometric torque applied to the handle was displayed as a cursor on the computer screen located in front of the subject. The cursor moved automatically across the screen from left to right at a constant velocity and subjects applied wrist flexion and extension muscle isometric contractions to move the cursor downward and upward respectively to track the double sine wave curve displayed on the screen. The second window provided visual feedback on the performance of the AT by displaying a series of white dots in a coordinate axis (Fig. 2D). Each dot represented the cumulative error (distance) in a single frame between the trace of the subject and the target. The lower the position of the dot on the y-axis, the lower the error between the double sine wave curve and their trace and thus the better the performance.

Practice of the AT during motor skill acquisition (blocks 1–3) consisted of 3×5 min blocks with 1 min of rest between blocks. The retention tests (blocks 4–6), performed 1 hour, 24 hours and 7 days after practice, consisted of 1 block of 5 min of practice each. In all trials, the sine wave curve was displayed in frames of 6 sec with 1 sec between frames in which the visual feedback (i.e. white dot) was provided. Performance was measured as the average root mean square value of the error distance between the subject's torque signal and the displayed target across all sampled data points in each frame. Subjects performed 42 frames every 5 min of practice (252 frames in total) in addition to the 10 trials performed at baseline.

### Data analysis

Assumptions of normality of the distribution for all variables were explored through histograms and normality plots and confirmed with the Shapiro-Wilk's normality test. Differences in exercise parameters (blood lactate concentration and perceived exertion) between exercise groups (PRE and POST) were compared with a student's t test. Performance among groups at baseline was compared with one-way analysis of variance (ANOVA). Differences in the acquisition and retention of the motor skill were analysed with two ANOVA. First, acquisition (blocks 1–3) was compared with two-way (block x group) repeated-measures ANOVA. Retention blocks (1 hour, 24 hours and 7 days) were compared with two-way (block x group) repeated-measures ANOVA. To analyze motor skill retention while factorizing for between-groups differences in skill level at the end of practice, performance of retention blocks was normalized to mean performance in the last block of practice (block 3). When the variances of the differences among different blocks (i.e. sphericity) were significantly different, the Greenhouse-Geisser correction was applied. When a significant main effect in the ANOVA was found, post-hoc pairwise comparisons for each block were performed with the student's t test. The level of statistical significance in the post-hoc comparisons was adjusted to a p≤0.017 using the Bonferroni's procedure (0.05/3). The effect size (*d*) for each retention test was calculated by dividing the difference between mean change scores of the experimental (PRE and POST) and control (CON) groups by the pooled standard deviation (SD). Unless otherwise stated, data were presented as means with standard error of the mean (SEM) and analyses performed with two-tailed probability tests with a statistical significance set at p<0.05. The reported p values are not adjusted.

## Results

The workload during the blocks of high intensity cycling exercise in both exercise groups ranged from 200W-315W. The mean (SEM) concentration of blood lactate after cycling was similar (t = −0.83; p = 0.72) between PRE [13.14 (0.82) mmol/l] and POST [12.72 (0.83) mmol/l)]. Similarly, no significant (t = 1.20; p = 0.93) differences in the subjective level of perceived exertion after cycling exercise were found between PRE [11.69 (0.67)] and POST [11.77 (0.63)].

**Figure 3:**
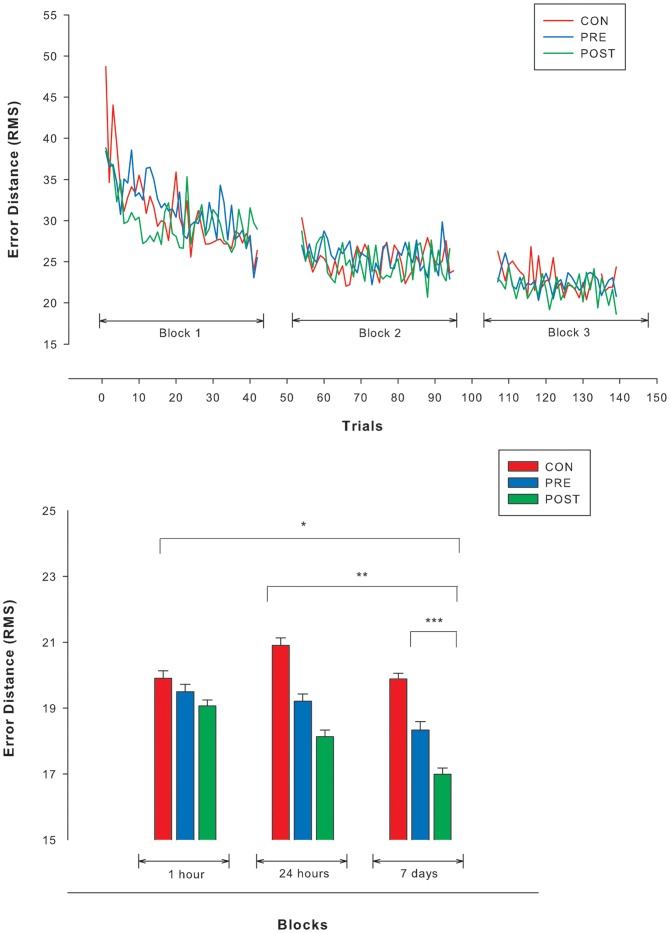
Performance of the visuomotor accuracy-tracking task (AT) during acquisition and retention. **A**. Performance was measured as the average root mean square (RMS) value of the error distance between the subject's torque signal and the displayed target. All groups showed similar performance in the rate of acquisition of the motor skill (p = 0.81) measured throughout the blocks 1–3 of practice. Each data point represents the average of each single trial. **B**. Performance was measured as the average root mean square (RMS) value of the error distance between the subject's torque signal and the displayed target. Overall differences among groups in the retention of the motor skill were found (*). The PRE and POST groups showed a better retention of the motor skill than the CON group 24 hours and 7 days after practice (**). POST also showed a better retention than PRE 7 days after practice (***). Data are presented as means and the error bars are standard error of the mean. All comparisons yielded a p value <0.001.

There were no significant differences among groups in performance of the AT at baseline [*F*(2,27)  = 1.76; p = 0.19]. Figure 3A shows the performance of all groups in the AT during motor skill acquisition (blocks 1–3). The repeated-measures ANOVA revealed similar rates in the acquisition of the motor skill during practice (blocks 1–3) [*F*(3.20,193.50)  = 0.34; p = 0.81]. In contrast, the second repeated-measures ANOVA showed significant differences among groups in the retention of the motor skill (Fig. 3B). Overall, both exercise groups performed better in the retention tests than controls [*F*(4,244)  = 16.51; p<0.001)]. Further post-hoc analyses confirmed a significant difference in retention between the exercise groups and CON, but only when retention was tested 24 hours (t = 5.50; p<0.001; *d* = 1.65 and t = 4.62; p<0.001; *d* = 1.55 for PRE and POST vs. CON respectively) and 7 days (t = 11.17; p<0.001; *d* = 1.26 and t = 3.96; p<0.001; *d* = 1.70 for PRE and POST vs. CON respectively) after practice. In contrast, differences between the exercise groups and CON in the short-term retention of the motor skill, assessed 1 hour after practice were not significant (t = −0.17; p = 0.86; *d* = 0.75 and t = 0.01; p = 0.99; *d* = 0.37 for PRE and POST vs. CON respectively). When retention of the motor skill between PRE and POST was compared, POST showed a better performance than PRE in all retention tests. However, differences were only significant when retention was tested 7 days after practice (t = 5.10; p<0.001; *d* = 0.44). Differences between PRE and POST in retention 1 hour (t = −0.18; p = 0.85; *d* = 0.37) and 24 hours (t = 0.97; p = 0.33; *d* = 0.11) after practice were not significant.

## Discussion

This study addressed two main questions: first, can a single bout of intense exercise optimize the acquisition and retention of a motor skill? Second, is the timing of exercise in relation to the practice of the motor task important to modulate the effects of acute exercise on motor memory and skill learning? With regards to the first question, our results suggest that a single bout of exercise does not have any significant effect on either the rate of acquisition nor the short-term retention of the motor skill assessed 1 hour after practice. In contrast, this type of exercise appears to improve motor learning through an optimization of motor memory. This was confirmed by the fact that both exercise groups showed a better retention of the motor skill than the control group, especially when retention was assessed 24 hours and 7 days after practice. With regards to the second question, we found timing of exercise not to be relevant when retention was assessed 1 hour and 24 hours after practice. However, timing of exercise appeared to be critical in modulating the effects of exercise on the long-term retention of motor memory. This was confirmed by the fact that, compared to the group that exercised before practice (PRE), the group that exercised after practice (POST) showed a better retention of the motor skill 7 days after practice. It is important to reiterate that the groups of the study were matched for age, gender and fitness level (Table 1) and that there were not significant differences in motor skill level at baseline. Since both exercise groups showed very similar levels of blood lactate and level of perceived exertion after exercise it is also conceivable that the intensity of exercise was very similar. It is therefore unlikely that these results could be confounded by differences in the characteristics of the subjects or the exercise protocol.

The first important finding of the present study concerns the null impact that acute exercise appeared to have on the acquisition of the motor skill in the group that exercised before practicing the AT (PRE). On the basis of previous data revealing enhanced performance in some cognitive tasks following acute exercise [24] we speculated that the bout of exercise might also enhance the rate of motor skill acquisition during practice. Winter et al. [33], for example, reported that the acquisition of novel words during an associative vocabulary-learning task was accelerated by 20% after 2×3 min of intense (>10 mmol/l lactate) treadmill running. In contrast, in our study, all groups showed similar improvements in motor skill acquisition (Fig. 3A). The positive influence of acute exercise on cognition is often manifested as facilitation during practice. However, gains in performance are highly selective to the type of cognitive task and the characteristics of the exercise protocol [24]. Our results indicate that the potential effects of acute exercise on the identification and encoding of procedural information during practice of the AT did not translate into immediate observable gains in motor skill acquisition. Perhaps the performance of an intense bout of exercise prior to a motor task that focused heavily on accuracy diluted the benefits of acute exercise on motor skill acquisition. Indeed, recent evidence suggests that even exercise at moderate intensity may have detrimental effects on accuracy performance [41]. It is not unlikely, for example, that while exercise had indeed optimized the rate of acquisition of the motor skill, the effects of underlying fatigue could have masked improvements in tracking accuracy during practice of the AT [42]. The coexistence of facilitative and hindering mechanisms during practice might well explain why intense exercise did not enhance nor impair motor skill acquisition.

In contrast to the null effects on acquisition, acute exercise optimized motor skill learning through an enhanced long-term retention of motor memory, as manifested in a better performance of both exercise groups in the retention of the motor skill 24 hours and 7 days after practice (Fig. 3B). This finding is important because delayed retention is a better indicator of learning than performance at or shortly after acquisition [35]. Although some investigations have revealed no significant [33], [43], [44] or even detrimental effects on memory [45], pooled together [24], the results of studies exploring how acute exercise modulates memory yield a positive, yet moderate, mean effect (*d* = 0.30; 95% CI  = 0.06,0.55) in cognitive tasks involving components of both short [17], [29], [31], [32], [46] and long-term memory [29], [34], [46]. These studies, however, have targeted cognitive tasks mainly requiring explicit forms of memory such as verbal memory (e.g. recall a series of words). This is, to our knowledge, the first study investigating the impact of acute exercise on motor memory and skill learning. Our findings add new evidence indicating that the benefits of this intervention on memory consolidation might generalize to other, more implicit, forms of memory and learning such as motor memory and skill learning. More studies will be required to determine if these promising results can be extrapolated to other motor skills and population groups.

It is unclear why, in contrast to the positive effects on long-term retention, acute exercise did not translate into a significantly better retention of the motor skill in the first retention test performed 1 hour after practice (Fig. 3B). One plausible explanation is that the proximity of the retention test to the intense exercise bout could have hindered the relatively short-term recall of the motor memory [29]. There is some evidence that high arousal levels at encoding may temporarily increase inhibition of retrieval during memory consolidation [47], which would have detrimental effects on short-term recall (45). Another possibility is that the representation of the motor memory was still undergoing consolidation when the first retention test was performed and thus the benefits of acute exercise on memory had not yet taken place. Inconsistencies between immediate and delayed retention tests are not unusual in motor skill learning studies. Recently, Kantak et al. [35] reviewed 41 motor skill learning studies that employed both immediate and delayed retention/transfer tests and found that 19 studies showed significant differences between groups only in the delayed retention tests [35]. These discrepancies possibly reflect different stages of the maturation of the motor memory when retention is assessed [48]. In our study, the apparent null effect of acute exercise on acquisition in addition to the optimization of the retention of the motor skill in the exercise groups reaffirm the importance of distinguishing learning from eventual improvements during or shortly after practice and emphasize the relevance of using delayed retention tests as a more reflective measure of motor skill learning [35].

Perhaps the most striking finding of the present study was the notable difference between exercise groups in the long-term retention of motor memory, as shown in the retention test performed 7 days after practice (Fig. 3B). These differences in the retention of the motor skill suggest that the timing of exercise in relation to motor practice is indeed an important factor regulating the effects of acute exercise on motor memory. We speculate that these differences could be due to the specific effects that acute exercise performed at different stages of the formation of memory has on the retention of the motor skill. Regardless of the time when it is performed, acute exercise appears to enhance motor memory. However, exercise performed before practice possibly does so by mainly optimizing the detection and encoding of procedural information during motor practice while exercise after practice improves exclusively its consolidation into long-lasting memory. In order to test this possibility, it had been interesting to investigate if the performance of two bouts of exercise, one before and a second after practice of the AT, would have led to an even better retention of the motor skill. More experimental studies are required to determine the specific temporal patterns regulating the effects of acute exercise on the formation of motor memory and to test the hypothesis that the enhancement of motor memory is produced by different mechanisms depending on the timing of exercise.

In our study, the bout of exercise performed after practice optimized the long-term retention of the motor skill more so than when exercise was performed before practice. This result conflicts with a recent study which assessed the effects of acute exercise performed at different time points on verbal memory [34]. In this study [34] the authors investigated the effects of 30 min of moderate aerobic exercise performed either before of after listening to two paragraphs (exposure) on the recall performance of these two paragraphs assessed 35 min after exposure. In comparison to a non-exercise control group, the group that exercised prior to exposure showed a significantly greater recall of words during retention although differences between exercise groups were not statistically significant. One possible explanation for the discrepancy between these results and our results might be in the differences in the characteristics of the task as well as the exercise protocol. It should be noted, however, that in the aforementioned study [34] the group that performed the exercise bout after exposure had to perform the retention test immediately after exercise. Thus, it is not unlikely that the proximity of the retention test to the exercise bout could have disproportionally increased neural noise thus impairing the recall of words at retention [49]. Unfortunately, the authors did not include further delayed retention tests, which could have been useful to assess verbal memory excluding the potential confounding effects of exercise on recall.

## Conclusions

A single bout of intense exercise performed immediately before or after practicing a motor task was sufficient to improve motor skill learning through a better long-term retention of the skill. The positive effects of acute exercise on motor memory appeared to be maximized when exercise was performed after practice, during the early stages of memory consolidation. The effects of acute exercise on the optimization of the storage and retrieval of motor memory may have important practical implications. Our results suggest that this intervention can be used to boost motor skill learning through an optimization of the consolidation of motor memory, which has significant perspectives in both sports and rehabilitation. More studies are needed to investigate the exact mechanisms by which acute exercise optimizes motor memory consolidation. Only then, we will be able to be accurate in the prescription of personalized exercise interventions to optimize the acquisition and retention of motor skills.
